# Supplementary material for: Research on the expansion, shrinkage properties and fracture evolution of red clay stabilised with phosphogypsum under dry-wet cycles
Source: PLoS One. 2024 Aug 20;19(8):e0308616. doi: 10.1371/journal.pone.0308616 (PMC11335147; doi:10.1371/journal.pone.0308616)
Supplement: S1 Data set — (DOCX) [file pone.0308616.s001.docx]

Research on the expansion, shrinkage properties and fracture evolution of red clay stabilised with phosphogypsum under dry-wet cycles

**Jinxiong Chen^1 ¶^, Kaisheng Chen^1^** **^*^**

1 School of Civil Engineering, Guizhou University, Guiyang, Guizhou Province, China

*[chen_kaisheng@163.com](mailto:chen_kaisheng@163.com)(KC)

¶ These authors also contributed equally to this work.

**Tip: For the sake of aesthetics, the experimental data in this paper are the results after the omission of %, the experimental data in the corresponding experimental results below the picture**

Tab 1. Basic physical indexes of red clay

| $\boldsymbol{\rho}$/g‧cm^-3^ | $\boldsymbol{\omega}$/% | $\boldsymbol{\omega}_{\boldsymbol{op}}$/% | $\boldsymbol{\rho}_{\boldsymbol{dmax}}$/g‧cm^-3^ | $\boldsymbol{W}_{\boldsymbol{L}}$/% | $\boldsymbol{W}_{\boldsymbol{P}}$/% | Cu | Cc |
| --- | --- | --- | --- | --- | --- | --- | --- |
| 1.76 | 60.03 | 30.24 | 1.46 | 82.13 | 43.02 | 10.63 | 1.085 |

Tab 2. Basic parameters of phosphogypsum

| Specific surface area  / m^2^‧kg^-1^ | Loss on ignition /% | Moisture content /% | Alkali content /% | Density  /g‧cm^-3^ | fineness  /% |
| --- | --- | --- | --- | --- | --- |
| 102 | 18.43 | 5.3 | 1.31 | 2.38 | 44.3 |

Tab 3. Chemical composition of phosphogypsum

| Ingredient | SO_3_ | CaO | SiO_2_ | P_2_O_5_ | Na_2_O | Al_2_O_3_ | Other |
| --- | --- | --- | --- | --- | --- | --- | --- |
| Mass fraction/% | 49.070 | 40.070 | 5.780 | 1.350 | 0.587 | 0.435 | 2.708 |

Tab 4. Test results of heavy metals and radioactivity in phosphogypsum

| Test items | | Standard limits | Result | Conclusion |
| --- | --- | --- | --- | --- |
| Heavy metal | Cu/mg‧L^-1^ | ≤100 | 0.157 | Qualified |
|  | Zn/mg‧L^-1^ | ≤100 | 0.051 | Qualified |
|  | Cd/mg‧L^-1^ | ≤1 | 0 | Qualified |
|  | Pb/mg‧L^-1^ | ≤5 | 0 | Qualified |
|  | Cr/mg‧L^-1^ | ≤15 | 0 | Qualified |
|  | As/mg‧L^-1^ | ≤5 | 0.0356 | Qualified |
|  | Hg/mg‧L^-1^ | ≤0.1 | 0.0005 | Qualified |
| Radioactivity | Ra-226/Bq‧kg^-1^ | — | 53.94 | — |
|  | TH-232/Bq‧kg^-1^ | — | 42.13 | — |
|  | K-40/Bq‧kg^-1^ | — | 52.95 | — |
|  | I_Ra_ | ≤1.0 | 0.3 | Qualified |
|  | I_γ_ | ≤1.0 | 0.3 | Qualified |

Tab 5. Basic parameters of cement

| Item | Index | Item | Index | Item | Index |
| --- | --- | --- | --- | --- | --- |
| 3d $f_{cf}$/MPa | 5.0 | Loss on ignition /% | 1.58 | Initial setting time /min | 302 |
| 28d $f_{cf}$/MPa | 6.7 | Alkali /% | 2.42 | Final setting time /min | 322 |
| 3d $f_{cu}$/MPa | 24.9 | Chloride ion /% | 0.018 | Stability | Qualified |
| 28d $f_{cu}$/MPa | 43.7 | Sulfur trioxide /% | 2.87 |  |  |

Tab 6. Optimal moisture content and maximum dry density of mixture under different proportion and cement content

| P:T | 4% C | | 6% C | | 8% C | |
| --- | --- | --- | --- | --- | --- | --- |
|  | OMC/% | MDD/g$\cdot$cm^-3^ | OMC/% | MDD/g$\cdot$cm^-3^ | OMC/% | MDD/g$\cdot$cm^-3^ |
| 1:1 | 21.3 | 1.559 | 21.8 | 1.555 | 20.8 | 1.569 |
| 1:2 | 22.5 | 1.535 | 23.1 | 1.549 | 21.8 | 1.556 |
| 1:3 | 23.46 | 1.519 | 24.1 | 1.538 | 22.9 | 1.543 |
| 1:4 | 24.36 | 1.451 | 25.6 | 1.511 | 24.5 | 1.499 |
| 1:5 | 26.0 | 1.459 | 26.7 | 1.495 | 25.7 | 1.504 |

Note: In this paper, C stands for cement, P stands for phosphogypsum, T stands for red clay, OMC stands for optimum moisture content, MDD stands for maximum dry density, and PCAS stands for image processing system.

Tab 7. Specimen Preparation

| Batch number | Compaction /% | C/% | Moisture content /% | P:T | Number of wet-dry cycles /times | Number of samples /pcs |
| --- | --- | --- | --- | --- | --- | --- |
| 1 | 90,92,93,94,95,96 | 6 | OMC | 1:1,1:2,1:31:4,1:5 | 1~7 | 30 |
| 2 | 93 | 4,6,8 | OMC | 1:1,1:2,1:3,1:4,1:5 | 1~7 | 15 |
| 3 | 93 | 6 | OMC-3%,OMC, OMC +3% | 1:1,1:2,1:3,1:4,1:5 | 1~7 | 15 |

|  |
| --- |

|  (a)P:T=1:1 |  (b)P:T=1:2 | |  (c)P:T=1:3 |
| --- | --- | --- | --- |
| (d)P:T=1:4 | | (e)P:T=1:5 | |

Fig 4. Relationship between absolute expansion and compaction

Raw data for Figure 4

|  |  | 1 | 2 | 3 | 4 | 5 | 6 | 7 |
| --- | --- | --- | --- | --- | --- | --- | --- | --- |
| (a) | 90 | 0.8 | 0.92 | 0.97 | 1.05 | 1.1 | 1.1 | 1.08 |
|  | 92 | 0.85 | 0.99 | 1.06 | 1.17 | 1.17 | 1.15 | 1.16 |
|  | 93 | 0.9 | 1.05 | 1.2 | 1.25 | 1.3 | 1.25 | 1.25 |
|  | 94 | 1.02 | 1.1 | 1.22 | 1.3 | 1.33 | 1.29 | 1.28 |
|  | 95 | 1.05 | 1.15 | 1.3 | 1.37 | 1.37 | 1.35 | 1.33 |
|  | 96 | 1.1 | 1.19 | 1.36 | 1.43 | 1.41 | 1.38 | 1.37 |
| (b) | 90 | 0.9 | 1.05 | 1.19 | 1.25 | 1.28 | 1.3 | 1.3 |
|  | 92 | 0.96 | 1.13 | 1.28 | 1.35 | 1.39 | 1.4 | 1.41 |
|  | 93 | 0.98 | 1.18 | 1.32 | 1.42 | 1.5 | 1.55 | 1.55 |
|  | 94 | 1.02 | 1.25 | 1.37 | 1.49 | 1.55 | 1.62 | 1.63 |
|  | 95 | 1.06 | 1.31 | 1.52 | 1.64 | 1.72 | 1.78 | 1.75 |
|  | 96 | 1.09 | 1.48 | 1.7 | 1.89 | 2 | 2.03 | 2.05 |
| (c) | 90 | 2.15 | 3.15 | 3.7 | 3.85 | 3.9 | 3.95 | 4.05 |
|  | 92 | 2.25 | 3.25 | 4.05 | 3.9 | 4.25 | 4.4 | 4.5 |
|  | 93 | 2.95 | 3.7 | 4.35 | 4.85 | 5.1 | 5.35 | 5.6 |
|  | 94 | 2.35 | 3.7 | 4.3 | 4.7 | 4.85 | 5.1 | 5.3 |
|  | 95 | 2.75 | 3.75 | 4.55 | 5.15 | 5.4 | 5.5 | 5.8 |
|  | 96 | 2.9 | 3.8 | 4.75 | 5.25 | 5.4 | 5.7 | 6 |
| (d) | 90 | 5.85 | 10.45 | 10.85 | 11.05 | 11 | 10.95 | 10.8 |
|  | 92 | 5.85 | 10.45 | 11.2 | 11.8 | 12.15 | 12.3 | 12.2 |
|  | 93 | 6.35 | 11.2 | 12.55 | 12.8 | 13.2 | 13.3 | 13.5 |
|  | 94 | 6.75 | 11.9 | 13 | 13.6 | 14.05 | 14.25 | 14.6 |
|  | 95 | 6.75 | 12.45 | 13.75 | 14.2 | 14.85 | 15.05 | 15.5 |
|  | 96 | 7.25 | 13.45 | 14.8 | 15.5 | 16.25 | 16.7 | 17.2 |
| (e) | 90 | 6.2 | 11.35 | 12.5 | 13.6 | 13.45 | 13.7 | 13.9 |
|  | 92 | 6.35 | 11.45 | 13.2 | 14.7 | 14.75 | 14.9 | 15.1 |
|  | 93 | 6.5 | 11.45 | 12.9 | 14.35 | 14.4 | 14.6 | 14.95 |
|  | 94 | 6.25 | 11.25 | 12.95 | 14.4 | 14.65 | 14.95 | 15.35 |
|  | 95 | 6.15 | 11.35 | 13.25 | 14.85 | 15.45 | 15.65 | 16.1 |
|  | 96 | 6.4 | 12.2 | 14.15 | 15.75 | 16.6 | 17.15 | 17.5 |

| (a)P:T=1:1 | (b)P:T=1:2 | | (c)P:T=1:3 |
| --- | --- | --- | --- |
|  (d)P:T=1:4 | | (e)P:T =1:5 | |

Fig 5. Absolute expansion rate versus number of wet-dry cycles

Raw data for Figure 5

|  |  | 90 | 92 | 93 | 94 | 95 | 96 |
| --- | --- | --- | --- | --- | --- | --- | --- |
| (a) | 1 | 0.8 | 0.85 | 0.9 | 1.02 | 1.05 | 1.1 |
|  | 2 | 0.92 | 0.99 | 1.05 | 1.1 | 1.15 | 1.19 |
|  | 3 | 0.97 | 1.06 | 1.2 | 1.22 | 1.3 | 1.36 |
|  | 4 | 1.05 | 1.17 | 1.25 | 1.3 | 1.37 | 1.43 |
|  | 5 | 1.1 | 1.17 | 1.3 | 1.33 | 1.37 | 1.41 |
|  | 6 | 1.1 | 1.15 | 1.25 | 1.29 | 1.35 | 1.38 |
|  | 7 | 1.08 | 1.16 | 1.25 | 1.28 | 1.33 | 1.37 |
| (b) | 1 | 0.9 | 0.96 | 0.98 | 1.02 | 1.06 | 1.09 |
|  | 2 | 1.05 | 1.13 | 1.18 | 1.25 | 1.31 | 1.48 |
|  | 3 | 1.19 | 1.28 | 1.32 | 1.37 | 1.52 | 1.7 |
|  | 4 | 1.25 | 1.35 | 1.42 | 1.49 | 1.64 | 1.89 |
|  | 5 | 1.28 | 1.39 | 1.5 | 1.55 | 1.72 | 2 |
|  | 6 | 1.3 | 1.4 | 1.55 | 1.62 | 1.78 | 2.03 |
|  | 7 | 1.3 | 1.41 | 1.55 | 1.63 | 1.75 | 2.05 |
| (c) | 1 | 2.15 | 2.25 | 2.35 | 2.75 | 2.9 | 2.95 |
|  | 2 | 3.15 | 3.25 | 3.7 | 3.75 | 3.8 | 3.7 |
|  | 3 | 3.7 | 4.05 | 4.3 | 4.55 | 4.75 | 4.35 |
|  | 4 | 3.85 | 3.9 | 4.7 | 5.15 | 5.25 | 4.85 |
|  | 5 | 3.9 | 4.25 | 4.85 | 5.4 | 5.4 | 5.1 |
|  | 6 | 3.95 | 4.4 | 5.1 | 5.5 | 5.7 | 5.35 |
|  | 7 | 4.05 | 4.5 | 5.3 | 5.8 | 6 | 5.6 |
| (d) | 1 | 5.85 | 5.85 | 6.35 | 6.75 | 6.75 | 7.25 |
|  | 2 | 10.45 | 10.45 | 11.2 | 11.9 | 12.45 | 13.45 |
|  | 3 | 10.85 | 11.2 | 12.55 | 13 | 13.75 | 14.8 |
|  | 4 | 11.05 | 11.8 | 12.8 | 13.6 | 14.2 | 15.5 |
|  | 5 | 11 | 12.15 | 13.2 | 14.05 | 14.85 | 16.25 |
|  | 6 | 10.95 | 12.3 | 13.3 | 14.25 | 15.05 | 16.7 |
|  | 7 | 10.8 | 12.2 | 13.5 | 14.6 | 15.5 | 17.2 |
| (e) | 1 | 6.2 | 6.35 | 6.5 | 6.25 | 6.15 | 6.4 |
|  | 2 | 11.35 | 11.45 | 11.45 | 11.25 | 11.35 | 12.2 |
|  | 3 | 12.5 | 13.2 | 12.9 | 12.95 | 13.25 | 14.15 |
|  | 4 | 13.6 | 14.7 | 14.35 | 14.4 | 14.85 | 15.75 |
|  | 5 | 13.45 | 14.75 | 14.4 | 14.65 | 15.45 | 16.6 |
|  | 6 | 13.7 | 14.9 | 14.6 | 14.95 | 15.65 | 17.15 |
|  | 7 | 13.9 | 15.1 | 14.95 | 15.35 | 16.1 | 17.5 |

| (a)P:T=1:1 | (b)P:T=1:2 | | (c)P:T=1:3 |
| --- | --- | --- | --- |
| (d)P:T=1:4 | | (e)P:T =1:5 | |

Fig 6. Relationship between absolute expansion rate and cement dosage

Raw data for Figure 6

|  |  | 1 | 2 | 3 | 4 | 5 | 6 | 7 |
| --- | --- | --- | --- | --- | --- | --- | --- | --- |
| (a) | 4 | 0.65 | 0.65 | 0.8 | 0.85 | 0.8 | 0.7 | 0.75 |
|  | 6 | 0.9 | 0.95 | 1 | 0.95 | 1 | 0.85 | 0.85 |
|  | 8 | 1.55 | 1.6 | 1.9 | 2.2 | 2.45 | 2.4 | 2.5 |
| (b) | 4 | 0.95 | 1.25 | 1.1 | 0.8 | 0.8 | 0.75 | 0.8 |
|  | 6 | 0.95 | 1.05 | 1.25 | 1.3 | 1.4 | 1.55 | 1.75 |
|  | 8 | 2.3 | 2.8 | 3.35 | 3.3 | 3.6 | 3.85 | 4.2 |
| (c) | 4 | 2 | 2.85 | 3.5 | 3.6 | 3.55 | 3.65 | 3.7 |
|  | 6 | 2.35 | 3.7 | 4.3 | 4.7 | 4.85 | 5.1 | 5.3 |
|  | 8 | 2.65 | 4 | 4.55 | 5.15 | 5.4 | 5.6 | 5.7 |
| (d) | 4 | 4.85 | 9.35 | 9.95 | 10 | 10.3 | 10.55 | 10.7 |
|  | 6 | 6.35 | 11.2 | 12.55 | 12.8 | 13.2 | 13.3 | 13.5 |
|  | 8 | 6.95 | 11.8 | 13.9 | 14.3 | 14.75 | 14.65 | 14.65 |
| (e) | 4 | 5.4 | 10.15 | 10.85 | 11.55 | 11.7 | 11.65 | 11.8 |
|  | 6 | 6.5 | 11.45 | 12.9 | 14.35 | 14.4 | 14.6 | 14.95 |
|  | 8 | 7.1 | 11.75 | 13.5 | 14.45 | 15 | 15.55 | 15.9 |

| (a)C 4% | (b)C 6% |  (b)C 8% |
| --- | --- | --- |

Fig 7. Relationship between absolute expansion rate and P:C

Raw data for Figure 7

|  |  | 1 | 2 | 3 | 4 | 5 | 6 | 7 |
| --- | --- | --- | --- | --- | --- | --- | --- | --- |
| (a) | 1:1 | 0.65 | 0.65 | 0.8 | 0.85 | 0.8 | 0.7 | 0.75 |
|  | 1:2 | 0.95 | 1.25 | 1.1 | 0.8 | 0.8 | 0.75 | 0.8 |
|  | 1:3 | 2 | 2.85 | 3.5 | 3.6 | 3.55 | 3.65 | 3.7 |
|  | 1:4 | 4.85 | 9.35 | 9.95 | 10 | 10.3 | 10.55 | 10.7 |
|  | 1:5 | 5.4 | 10.15 | 10.85 | 11.55 | 11.7 | 11.65 | 11.8 |
| (b) | 1:1 | 0.9 | 0.95 | 1 | 0.95 | 1 | 0.85 | 0.85 |
|  | 1:2 | 0.95 | 1.05 | 1.25 | 1.3 | 1.4 | 1.55 | 1.75 |
|  | 1:3 | 2.35 | 3.7 | 4.3 | 4.7 | 4.85 | 5.1 | 5.3 |
|  | 1:4 | 6.35 | 11.2 | 12.55 | 12.8 | 13.2 | 13.3 | 13.5 |
|  | 1:5 | 6.5 | 11.45 | 12.9 | 14.35 | 14.4 | 14.6 | 14.95 |
| (c) | 1:1 | 1.55 | 1.6 | 1.9 | 2.2 | 2.45 | 2.4 | 2.5 |
|  | 1:2 | 2.3 | 2.8 | 3.35 | 3.3 | 3.6 | 3.85 | 4.2 |
|  | 1:3 | 2.65 | 4 | 4.55 | 5.15 | 5.4 | 5.6 | 5.7 |
|  | 1:4 | 6.95 | 11.8 | 13.9 | 14.3 | 14.75 | 14.65 | 14.65 |
|  | 1:5 | 7.1 | 11.75 | 13.5 | 14.45 | 15 | 15.55 | 15.9 |

| (a)P:T=1:1 | (b)P:T=1:3 | (c)P:T=1:5 |
| --- | --- | --- |

Fig 8. Relationship between absolute expansion and initial moisture content

Raw data for Figure 8

|  | 1 | 2 | 3 | 4 | 5 | 6 | 7 |
| --- | --- | --- | --- | --- | --- | --- | --- |
| (a) | 1.45 | 2.05 | 2.15 | 2.4 | 2.5 | 2.6 | 2.7 |
|  | 0.9 | 0.95 | 1 | 0.95 | 1 | 0.85 | 0.85 |
|  | 0.65 | 0.55 | 0.35 | 0.25 | 0.1 | -0.05 | -0.3 |
| (b) | 2.5 | 4.75 | 5.9 | 6.6 | 7 | 7.5 | 7.85 |
|  | 2.35 | 3.7 | 4.3 | 4.7 | 4.85 | 5.1 | 5.3 |
|  | 2.05 | 2.45 | 2.95 | 2.95 | 2.95 | 2.95 | 2.85 |
| (c) | 6.95 | 14.5 | 16.85 | 18.8 | 19.8 | 20.4 | 20.65 |
|  | 6.5 | 11.45 | 12.9 | 14.35 | 14.4 | 14.6 | 14.95 |
|  | 4.6 | 7.55 | 8.75 | 8.95 | 9.1 | 9.2 | 9.15 |

| (a)P:T=1:1 | (b)P:T=1:2 | | (c)P:T=1:3 |
| --- | --- | --- | --- |
| (d)P:T=1:4 | | (e)P:T =1:5 | |

Fig 9. Relationship between Absolute Expansion and Compaction

Raw data for Figure 9

|  |  | 1 | 2 | 3 | 4 | 5 | 6 | 7 |
| --- | --- | --- | --- | --- | --- | --- | --- | --- |
| (a) | 90 | -0.45 | -0.25 | -0.09 | 0.07 | 0.19 | 0.21 | 0.25 |
|  | 92 | -0.4 | -0.23 | -0.03 | 0.16 | 0.25 | 0.27 | 0.27 |
|  | 93 | -0.35 | -0.19 | 0.11 | 0.25 | 0.28 | 0.31 | 0.32 |
|  | 94 | -0.32 | -0.15 | 0.15 | 0.28 | 0.33 | 0.35 | 0.35 |
|  | 95 | -0.28 | -0.1 | 0.22 | 0.32 | 0.42 | 0.41 | 0.38 |
|  | 96 | -0.2 | -0.06 | 0.28 | 0.37 | 0.43 | 0.46 | 0.49 |
| (b) | 90 | -0.65 | -0.6 | -0.6 | -0.45 | -0.05 | 0.35 | 0.45 |
|  | 92 | -0.7 | -0.25 | -0.1 | 0.05 | 0.5 | 0.7 | 0.55 |
|  | 93 | -0.9 | -0.2 | -0.05 | 0.2 | 0.6 | 0.95 | 0.85 |
|  | 94 | -0.65 | 0.2 | 0.25 | 0.65 | 0.8 | 1 | 1.1 |
|  | 95 | -0.85 | 0.1 | 0.25 | 0.25 | 0.45 | 0.85 | 0.95 |
|  | 96 | -0.85 | -0.1 | 0.4 | 0.85 | 1.1 | 1.45 | 1.55 |
| (c) | 90 | -0.05 | 1.55 | 2.4 | 2.8 | 3 | 3.25 | 3.5 |
|  | 92 | 0 | 1.85 | 2.5 | 3 | 3.45 | 3.6 | 3.75 |
|  | 93 | 0.3 | 2.2 | 3.3 | 3.6 | 4.05 | 4.4 | 4.6 |
|  | 94 | 0.35 | 2.4 | 3.6 | 4.1 | 4.5 | 4.85 | 5.15 |
|  | 95 | 0.5 | 2.55 | 3.75 | 4.15 | 4.6 | 5.1 | 5.45 |
|  | 96 | 0.2 | 2.3 | 3.35 | 3.8 | 4.25 | 4.6 | 5 |
| (d) | 90 | 2.35 | 8.1 | 9.55 | 9.7 | 9.9 | 9.85 | 9.85 |
|  | 92 | 2.2 | 8.45 | 10.2 | 10.65 | 11.2 | 11.2 | 11.25 |
|  | 93 | 2.9 | 9.4 | 11.1 | 11.65 | 12.3 | 12.4 | 12.6 |
|  | 94 | 3.55 | 10.25 | 11.8 | 12.4 | 13 | 13.45 | 13.65 |
|  | 95 | 3.8 | 10.95 | 12.3 | 13.1 | 13.75 | 14.2 | 14.7 |
|  | 96 | 4.6 | 11.9 | 13.45 | 14.45 | 15.35 | 15.9 | 16.45 |
| (e) | 90 | 3 | 9.35 | 11.25 | 12.15 | 12.25 | 12.6 | 12.85 |
|  | 92 | 3.25 | 9.75 | 12.15 | 13.2 | 13.5 | 13.85 | 14 |
|  | 93 | 3.05 | 9.6 | 11.6 | 12.95 | 13.25 | 13.55 | 13.95 |
|  | 94 | 2.65 | 9.55 | 11.65 | 13 | 13.5 | 13.95 | 14.25 |
|  | 95 | 2.6 | 9.65 | 12 | 13.85 | 14.25 | 14.65 | 15.15 |
|  | 96 | 3.15 | 10.3 | 12.85 | 14.8 | 15.6 | 16.1 | 16.6 |

| (a)P:T=1:1 | (b)P:T=1:2 | | (c)P:T=1:3 |
| --- | --- | --- | --- |
| (d)P:T=1:4 | | (e)P:T =1:5 | |

Fig 10. Relationship between absolute shrinkage and number of wet-dry cycles

Raw data for Figure 10

|  |  | 90 | 92 | 93 | 94 | 95 | 96 |
| --- | --- | --- | --- | --- | --- | --- | --- |
| (a) | 1 | -0.45 | -0.4 | -0.35 | -0.32 | -0.28 | -0.2 |
|  | 2 | -0.25 | -0.23 | -0.19 | -0.15 | -0.1 | -0.06 |
|  | 3 | -0.09 | -0.03 | 0.11 | 0.15 | 0.22 | 0.28 |
|  | 4 | 0.07 | 0.16 | 0.25 | 0.28 | 0.32 | 0.37 |
|  | 5 | 0.19 | 0.25 | 0.28 | 0.33 | 0.42 | 0.43 |
|  | 6 | 0.21 | 0.27 | 0.31 | 0.35 | 0.41 | 0.46 |
|  | 7 | 0.25 | 0.27 | 0.32 | 0.35 | 0.38 | 0.49 |
| (b) | 1 | -0.65 | -0.7 | -0.9 | -0.65 | -0.85 | -0.85 |
|  | 2 | -0.6 | -0.25 | -0.2 | 0.2 | 0.1 | -0.1 |
|  | 3 | -0.6 | -0.1 | -0.05 | 0.25 | 0.25 | 0.4 |
|  | 4 | -0.45 | 0.05 | 0.2 | 0.65 | 0.25 | 0.85 |
|  | 5 | -0.05 | 0.5 | 0.6 | 0.8 | 0.45 | 1.1 |
|  | 6 | 0.35 | 0.7 | 0.95 | 1 | 0.85 | 1.45 |
|  | 7 | 0.45 | 0.55 | 0.85 | 1.1 | 0.95 | 1.55 |
| (c) | 1 | -0.05 | 0 | 0.3 | 0.35 | 0.5 | 0.2 |
|  | 2 | 1.55 | 1.85 | 2.2 | 2.4 | 2.55 | 2.3 |
|  | 3 | 2.4 | 2.5 | 3.3 | 3.6 | 3.75 | 3.35 |
|  | 4 | 2.8 | 3 | 3.6 | 4.1 | 4.15 | 3.8 |
|  | 5 | 3 | 3.45 | 4.05 | 4.5 | 4.6 | 4.25 |
|  | 6 | 3.25 | 3.6 | 4.4 | 4.85 | 5.1 | 4.6 |
|  | 7 | 3.5 | 3.75 | 4.6 | 5.15 | 5.45 | 5 |
| (d) | 1 | 2.35 | 2.2 | 2.9 | 3.55 | 3.8 | 4.6 |
|  | 2 | 8.1 | 8.45 | 9.4 | 10.25 | 10.95 | 11.9 |
|  | 3 | 9.55 | 10.2 | 11.1 | 11.8 | 12.3 | 13.45 |
|  | 4 | 9.7 | 10.65 | 11.65 | 12.4 | 13.1 | 14.45 |
|  | 5 | 9.9 | 11.2 | 12.3 | 13 | 13.75 | 15.35 |
|  | 6 | 9.85 | 11.2 | 12.4 | 13.45 | 14.2 | 15.9 |
|  | 7 | 9.85 | 11.25 | 12.6 | 13.65 | 14.7 | 16.45 |
| (e) | 1 | 3 | 3.25 | 3.05 | 2.65 | 2.6 | 3.15 |
|  | 2 | 9.35 | 9.75 | 9.6 | 9.55 | 9.65 | 10.3 |
|  | 3 | 11.25 | 12.15 | 11.6 | 11.65 | 12 | 12.85 |
|  | 4 | 12.15 | 13.2 | 12.95 | 13 | 13.85 | 14.8 |
|  | 5 | 12.25 | 13.5 | 13.25 | 13.5 | 14.25 | 15.6 |
|  | 6 | 12.6 | 13.85 | 13.55 | 13.95 | 14.65 | 16.1 |
|  | 7 | 12.85 | 14 | 13.95 | 14.25 | 15.15 | 16.6 |

| (a)P:T=1:1 | (b)P:T=1:2 | | (c)P:T=1:3 |
| --- | --- | --- | --- |
| (d)P:T=1:4 | | (e)P:T=1:5 | |

Fig 11. Relationship between absolute shrinkage and cement dosage

Raw data for Figure 11

|  |  | 1 | 2 | 3 | 4 | 5 | 6 | 7 |
| --- | --- | --- | --- | --- | --- | --- | --- | --- |
| (a) | 4 | -0.55 | -0.25 | -0.1 | 0.05 | 0.15 | 0.2 | 0.25 |
|  | 6 | -0.35 | -0.4 | -0.25 | 0.15 | 0.15 | 0.2 | 0.3 |
|  | 8 | 0.2 | 0.45 | 0.85 | 1.25 | 1.6 | 1.85 | 1.9 |
| (b) | 4 | -0.75 | -0.2 | -0.15 | -0.1 | 0 | 0.1 | 0.15 |
|  | 6 | -0.9 | -0.2 | -0.05 | 0.2 | 0.6 | 0.95 | 0.85 |
|  | 8 | 0.25 | 1.4 | 1.75 | 2.15 | 2.55 | 3 | 3.5 |
| (c) | 4 | 0 | 1.45 | 2.35 | 2.5 | 2.8 | 3 | 3.05 |
|  | 6 | 0.3 | 2.2 | 3.3 | 3.6 | 4.05 | 4.4 | 4.6 |
|  | 8 | 0.2 | 2.3 | 3.25 | 3.85 | 4.3 | 4.7 | 4.85 |
| (d) | 4 | 1.9 | 7.4 | 8.6 | 8.95 | 9.35 | 9.8 | 9.9 |
|  | 6 | 2.9 | 9.4 | 11.1 | 11.65 | 12.3 | 12.4 | 12.6 |
|  | 8 | 3 | 9.9 | 12.3 | 12.95 | 13.5 | 13.6 | 13.75 |
| (e) | 4 | 2.3 | 8.1 | 9.4 | 10.35 | 10.45 | 10.7 | 10.9 |
|  | 6 | 3.05 | 9.6 | 11.6 | 12.95 | 13.25 | 13.55 | 13.95 |
|  | 8 | 3.05 | 9.6 | 12.05 | 13.15 | 14.1 | 14.6 | 14.95 |

| (a)C 4% | (b)C 6% | (c)C 8% |
| --- | --- | --- |

Fig 12. Fig 12. Relationship between absolute shrinkage and P:T

Raw data for Figure 12

|  |  | 1 | 2 | 3 | 4 | 5 | 6 | 7 |
| --- | --- | --- | --- | --- | --- | --- | --- | --- |
| (a) | 1:1 | -0.55 | -0.25 | -0.1 | 0.05 | 0.15 | 0.2 | 0.25 |
|  | 1:2 | -0.75 | -0.2 | -0.15 | -0.1 | 0 | 0.1 | 0.15 |
|  | 1:3 | 0 | 1.45 | 2.35 | 2.5 | 2.8 | 3 | 3.05 |
|  | 1:4 | 1.9 | 7.4 | 8.6 | 8.95 | 9.35 | 9.8 | 9.9 |
|  | 1:5 | 2.3 | 8.1 | 9.4 | 10.35 | 10.45 | 10.7 | 10.9 |
| (b) | 1:1 | 0.9 | 0.95 | 1 | 0.95 | 1 | 0.85 | 0.85 |
|  | 1:2 | 0.95 | 1.05 | 1.25 | 1.3 | 1.4 | 1.55 | 1.75 |
|  | 1:3 | 2.35 | 3.7 | 4.3 | 4.7 | 4.85 | 5.1 | 5.3 |
|  | 1:4 | 6.35 | 11.2 | 12.55 | 12.8 | 13.2 | 13.3 | 13.5 |
|  | 1:5 | 6.5 | 11.45 | 12.9 | 14.35 | 14.4 | 14.6 | 14.95 |
| (c) | 1:1 | 1.55 | 1.6 | 1.9 | 2.2 | 2.45 | 2.4 | 2.5 |
|  | 1:2 | 2.3 | 2.8 | 3.35 | 3.3 | 3.6 | 3.85 | 4.2 |
|  | 1:3 | 2.65 | 4 | 4.55 | 5.15 | 5.4 | 5.6 | 5.7 |
|  | 1:4 | 6.95 | 11.8 | 13.9 | 14.3 | 14.75 | 14.65 | 14.65 |
|  | 1:5 | 7.1 | 11.75 | 13.5 | 14.45 | 15 | 15.55 | 15.9 |

| (a)P:T=1:3 | (b)P:T=1:4 | (c)P:T=1:5 |
| --- | --- | --- |

Fig 13. 5.2.5 Relationship between absolute shrinkage rate and initial moisture content

Raw data for Figure 13

|  | 1 | 2 | 3 | 4 | 5 | 6 | 7 |
| --- | --- | --- | --- | --- | --- | --- | --- |
| (a) | 0.35 | 1.1 | 1.25 | 1.75 | 2.05 | 2.1 | 2.25 |
|  | -0.35 | -0.4 | -0.25 | 0.15 | 0.15 | 0.2 | 0.3 |
|  | -0.6 | -0.75 | -0.95 | -0.75 | -0.85 | -0.85 | -0.8 |
| (b) | 0.95 | 3.45 | 4.8 | 5.55 | 6.25 | 6.85 | 7.25 |
|  | 0.3 | 2.2 | 3.3 | 3.6 | 4.05 | 4.4 | 4.6 |
|  | -0.25 | 0.6 | 1.3 | 1.9 | 1.95 | 2.05 | 2.05 |
| (c) | 5.45 | 13.15 | 15.65 | 17.9 | 18.95 | 19.55 | 19.9 |
|  | 3.05 | 9.6 | 11.6 | 12.95 | 13.25 | 13.55 | 13.95 |
|  | 0.65 | 5.35 | 7.05 | 7.7 | 8 | 8.2 | 8.3 |

|  |  |  |  | |  |  |  |
| --- | --- | --- | --- | --- | --- | --- | --- |
| (a)P:T=1:4 | | | | (b)P:T=1:5 | | | |
| Fig 16. Relationship between fracture ratio and number of dry-wet cycles  Raw data for table 16 | | | | | | | |

|  |  | 90 | 92 | 93 | 94 | 95 | 96 |
| --- | --- | --- | --- | --- | --- | --- | --- |
| (a) | 1 | 3.2 | 2.85 | 2.46 | 2.13 | 1.97 | 1.02 |
|  | 2 | 3.77 | 3.56 | 3.41 | 2.74 | 2.54 | 1.89 |
|  | 3 | 4.22 | 4.17 | 4.05 | 3.66 | 3.21 | 2.66 |
|  | 4 | 4.69 | 4.58 | 4.55 | 4.35 | 3.82 | 3.24 |
|  | 5 | 5.04 | 4.93 | 4.88 | 4.76 | 3.93 | 3.56 |
|  | 6 | 5.46 | 5.32 | 5.2 | 5.08 | 4.21 | 3.79 |
|  | 7 | 5.77 | 5.6 | 5.46 | 5.31 | 4.43 | 3.96 |
| (b) | 1 | 4.17 | 3.69 | 3.26 | 2.94 | 2.13 | 1.23 |
|  | 2 | 4.61 | 4.29 | 3.79 | 3.77 | 2.29 | 2.03 |
|  | 3 | 4.99 | 4.66 | 4.35 | 4.01 | 3.22 | 2.96 |
|  | 4 | 5.32 | 4.91 | 4.77 | 4.56 | 3.89 | 3.56 |
|  | 5 | 5.60 | 5.44 | 5.25 | 5.07 | 4.33 | 3.92 |
|  | 6 | 5.82 | 5.69 | 5.49 | 5.33 | 4.52 | 4.11 |
|  | 7 | 5.92 | 5.85 | 5.64 | 5.49 | 4.74 | 4.37 |

| (a)P:T=1:4 | (b)P:T=1:5 |
| --- | --- |

Fig 17. Relationship between fracture ratio and compaction degree

Raw data for Figure 17

|  |  | 1 | 2 | 3 | 4 | 5 | 6 | 7 |
| --- | --- | --- | --- | --- | --- | --- | --- | --- |
| (a) | 90 | 3.2 | 3.77 | 4.22 | 4.69 | 5.04 | 5.46 | 5.77 |
|  | 92 | 2.85 | 3.56 | 4.17 | 4.58 | 4.93 | 5.32 | 5.6 |
|  | 93 | 2.46 | 3.41 | 4.05 | 4.55 | 4.88 | 5.2 | 5.46 |
|  | 94 | 2.13 | 2.74 | 3.66 | 4.35 | 4.76 | 5.08 | 5.31 |
|  | 95 | 1.97 | 2.54 | 3.21 | 3.82 | 3.93 | 4.21 | 4.43 |
|  | 96 | 1.02 | 1.89 | 2.66 | 3.24 | 3.56 | 3.79 | 3.96 |
| (b) | 90 | 4.17 | 4.61 | 4.99 | 5.32 | 5.60 | 5.82 | 5.92 |
|  | 92 | 3.69 | 4.29 | 4.66 | 4.91 | 5.44 | 5.69 | 5.85 |
|  | 93 | 3.26 | 3.79 | 4.35 | 4.77 | 5.25 | 5.49 | 5.64 |
|  | 94 | 2.94 | 3.77 | 4.01 | 4.56 | 5.07 | 5.33 | 5.49 |
|  | 95 | 2.13 | 2.29 | 3.22 | 3.89 | 4.33 | 4.52 | 4.74 |
|  | 96 | 1.23 | 2.03 | 2.96 | 3.56 | 3.92 | 4.11 | 4.37 |

| (a)90% compaction | (b)93% compaction | (c)96% compaction |
| --- | --- | --- |

Fig 18. Relationship between fracture rate and P: T

Raw data for Figure 18

| (a) | 1:3 | 0.034 | 0.25 | 0.36 | 0.48 | 0.61 | 0.69 | 0.75 |
| --- | --- | --- | --- | --- | --- | --- | --- | --- |
|  | 1:4 | 3.2 | 3.77 | 4.22 | 4.69 | 5.04 | 5.46 | 5.77 |
|  | 1:5 | 4.17 | 4.61 | 4.99 | 5.32 | 5.60 | 5.82 | 5.92 |
| (b) | 1:3 | 0.026 | 0.18 | 0.31 | 0.39 | 0.47 | 0.54 | 0.58 |
|  | 1:4 | 2.46 | 3.41 | 4.05 | 4.55 | 4.88 | 5.2 | 5.46 |
|  | 1:5 | 3.26 | 3.79 | 4.35 | 4.77 | 5.25 | 5.49 | 5.64 |
| (c) | 1:3 | 0.02 | 0.13 | 0.19 | 0.24 | 0.29 | 0.36 | 0.44 |
|  | 1:4 | 1.02 | 1.89 | 2.66 | 3.24 | 3.56 | 3.79 | 3.96 |
|  | 1:5 | 1.23 | 2.03 | 2.96 | 3.56 | 3.92 | 4.11 | 4.37 |

| (a)P:T=1:4 | (b)P:T=1:5 |
| --- | --- |

Fig 19. Relationship between fracture ratio and cement content

Raw data for Figure 19

|  |  | 1 | 2 | 3 | 4 | 5 | 6 | 7 |
| --- | --- | --- | --- | --- | --- | --- | --- | --- |
| (a) | 4 | 2.81 | 3.76 | 4.4 | 4.9 | 5.23 | 5.55 | 5.81 |
|  | 6 | 2.46 | 3.41 | 4.05 | 4.55 | 4.88 | 5.2 | 5.46 |
|  | 8 | 2.03 | 2.98 | 3.62 | 4.12 | 4.45 | 4.77 | 5.03 |
| (b) | 4 | 3.69 | 4.22 | 4.78 | 5.2 | 5.68 | 5.92 | 6.07 |
|  | 6 | 3.26 | 3.79 | 4.35 | 4.77 | 5.25 | 5.49 | 5.64 |
|  | 8 | 2.99 | 3.52 | 4.08 | 4.5 | 4.98 | 5.22 | 5.37 |

| (a)P:T=1:4 | (b)P:T=1:5 |
| --- | --- |

Fig 20. Relationship between fracture ratio and initial water content

Raw data for Figure 20

| (a) | 23 | 2.61 | 3.53 | 3.77 | 4.71 | 5.02 | 5.43 | 5.61 |
| --- | --- | --- | --- | --- | --- | --- | --- | --- |
|  | 26 | 2.46 | 3.41 | 3.56 | 4.55 | 4.88 | 5.2 | 5.46 |
|  | 29 | 2.34 | 3.2 | 3.41 | 4.36 | 4.63 | 4.95 | 5.27 |
| (b) | 23 | 3.51 | 4.05 | 4.53 | 4.98 | 5.47 | 5.71 | 5.82 |
|  | 26 | 3.26 | 3.79 | 4.35 | 4.77 | 5.25 | 5.49 | 5.64 |
|  | 29 | 3.02 | 3.47 | 4.22 | 4.51 | 4.97 | 5.22 | 5.41 |

Tab 8 Fitting results

| Coefficient | $\delta_{ae}$ | | $\delta_{as}$ | |
| --- | --- | --- | --- | --- |
| a | 15.01025 | | 13.2323 | |
| b | -0.4023 | | -0.12707 | |
| c | -0.08332 | | -0.07336 | |
| d | 0.02978 | | 0.0184 | |
| e | -670.59986 | | -592.44555 | |
| R2 | 0.94516 | | 0.84533 | |
| 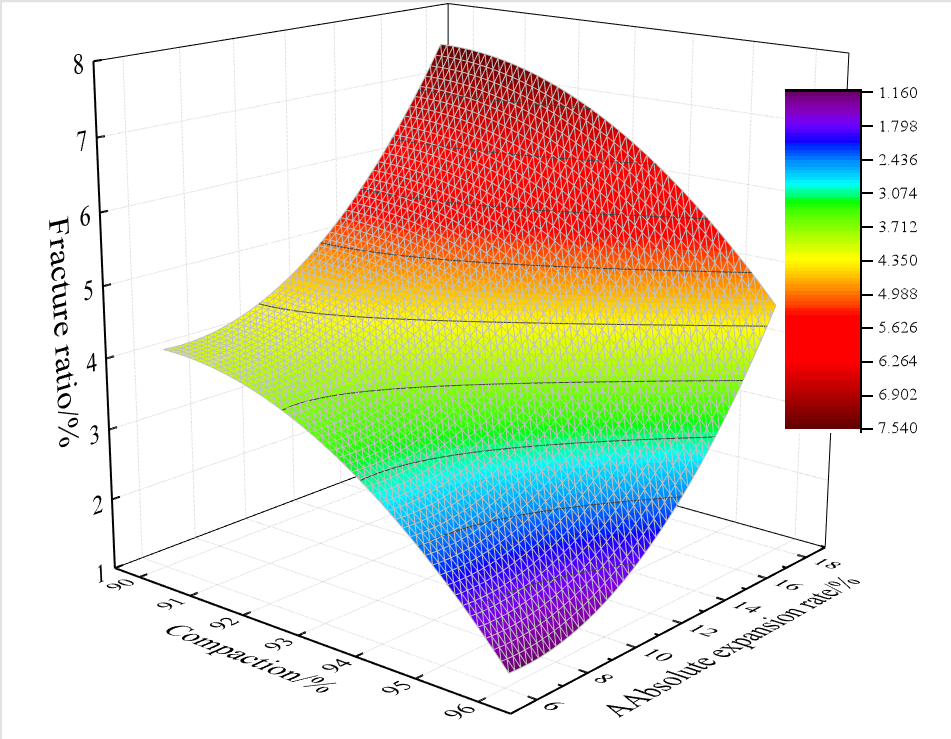(a) Absolute expansion rate fitting | | 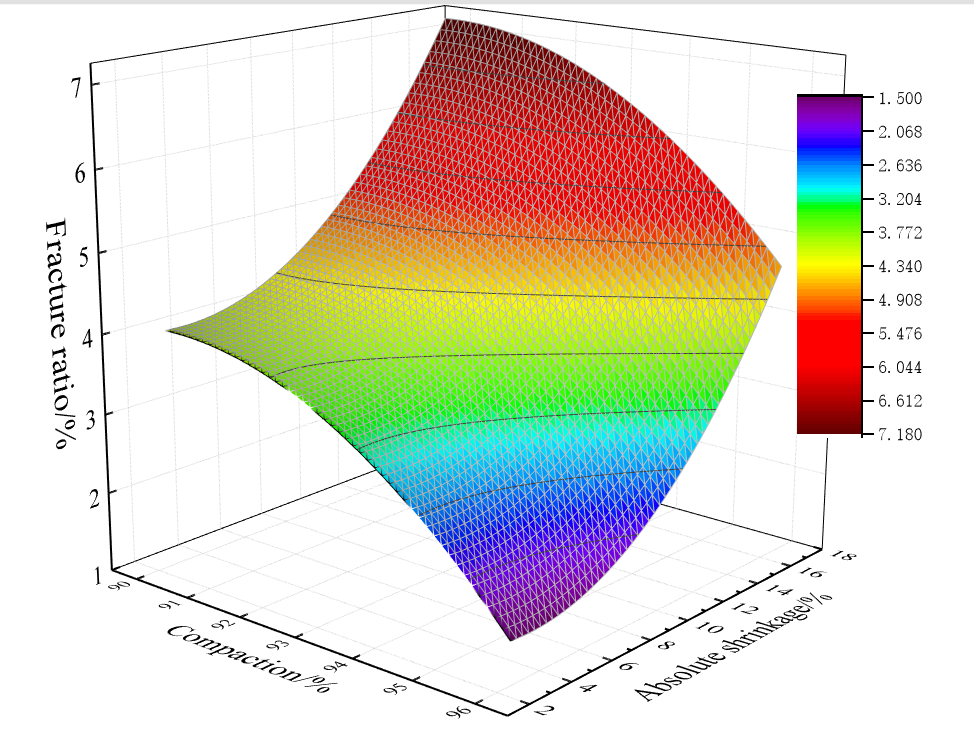(b) Absolute shrinkage fitting | |  |

Fig 21. Fitting results

Fig 21. Data used for fitting

| Compaction | Absolute expansion rate | Fracture ratio | Compaction | Absolute shrinkage rate | Fracture ratio |
| --- | --- | --- | --- | --- | --- |
| 1 wet-dry cycle | | | | | |
| 90 | 6.2 | 4.17 | 90 | 3 | 4.17 |
| 92 | 6.35 | 3.69 | 92 | 3.25 | 3.69 |
| 93 | 6.5 | 3.26 | 93 | 3.05 | 3.26 |
| 94 | 6.25 | 2.94 | 95 | 2.65 | 2.94 |
| 95 | 6.15 | 2.13 | 95 | 2.6 | 2.13 |
| 96 | 6.4 | 1.23 | 96 | 3.15 | 1.23 |
| 2 wet-dry cycles | | | | | |
| 90 | 11.35 | 4.61 | 90 | 9.35 | 4.61 |
| 92 | 11.45 | 4.29 | 92 | 9.75 | 4.29 |
| 93 | 11.45 | 3.79 | 93 | 9.6 | 3.79 |
| 94 | 11.25 | 3.77 | 95 | 9.55 | 3.77 |
| 95 | 11.35 | 2.29 | 95 | 9.65 | 2.29 |
| 96 | 12.2 | 2.03 | 96 | 10.3 | 2.03 |
| 3 wet-dry cycles | | | | | |
| 90 | 12.5 | 4.99 | 90 | 11.25 | 4.99 |
| 92 | 13.2 | 4.66 | 92 | 12.15 | 4.66 |
| 93 | 12.9 | 4.35 | 93 | 11.6 | 4.35 |
| 94 | 12.95 | 4.01 | 95 | 11.65 | 4.01 |
| 95 | 13.25 | 3.22 | 95 | 12 | 3.22 |
| 96 | 14.15 | 2.96 | 96 | 12.85 | 2.96 |
| 4 wet-dry cycles | | | | | |
| 90 | 13.6 | 5.32 | 90 | 12.15 | 5.32 |
| 92 | 14.7 | 4.91 | 92 | 13.2 | 4.91 |
| 93 | 14.35 | 4.77 | 93 | 12.95 | 4.77 |
| 94 | 14.4 | 4.56 | 95 | 13 | 4.56 |
| 95 | 14.85 | 3.89 | 95 | 13.85 | 3.89 |
| 96 | 15.75 | 3.56 | 96 | 14.8 | 3.56 |
| 5 wet-dry cycles | | | | | |
| 90 | 13.45 | 5.6 | 90 | 12.25 | 5.6 |
| 92 | 14.75 | 5.44 | 92 | 13.5 | 5.44 |
| 93 | 14.4 | 5.25 | 93 | 13.25 | 5.25 |
| 94 | 14.65 | 5.07 | 95 | 13.5 | 5.07 |
| 95 | 15.45 | 4.33 | 95 | 14.25 | 4.33 |
| 96 | 16.6 | 3.92 | 96 | 15.6 | 3.92 |
| 6 wet-dry cycles | | | | | |
| 90 | 13.7 | 5.82 | 90 | 12.6 | 5.82 |
| 92 | 14.9 | 5.69 | 92 | 13.85 | 5.69 |
| 93 | 14.6 | 5.49 | 93 | 13.55 | 5.49 |
| 94 | 14.95 | 5.33 | 95 | 13.95 | 5.33 |
| 95 | 15.65 | 4.52 | 95 | 14.65 | 4.52 |
| 96 | 17.15 | 4.11 | 96 | 16.1 | 4.11 |
| 7 wet-dry cycles | | | | | |
| 90 | 13.9 | 5.92 | 90 | 12.85 | 5.92 |
| 92 | 15.1 | 5.85 | 92 | 14 | 5.85 |
| 93 | 14.95 | 5.64 | 93 | 13.95 | 5.64 |
| 94 | 15.35 | 5.49 | 95 | 14.25 | 5.49 |
| 95 | 16.1 | 4.74 | 95 | 15.15 | 4.74 |
| 96 | 17.5 | 4.37 | 96 | 16.6 | 4.37 |
