# Supplementary material for: Research on the expansion, shrinkage properties and fracture evolution of red clay stabilised with phosphogypsum under dry-wet cycles
Source: PLoS One. 2024 Aug 20;19(8):e0308616. doi: 10.1371/journal.pone.0308616 (PMC11335147; doi:10.1371/journal.pone.0308616)
Supplement: Attachment — Submitted filename: Response to Reviewers.docx [file pone.0308616.s002.docx]

# **Response to Reviewers**

Dear Academic Editors and Reviewers:

Thank you very much for your valuable comments on the manuscript entitled “Research on the expansion, shrinkage properties and fracture evolution of red clay stabilised with phosphogypsum under dry-wet cycles”. In order to further meet the journal's publication criteria, we have scrutinized the linguistic presentation of the manuscript and completed the corresponding revisions, and the revised manuscript has been submitted to the journal.
